# Dual Infection of an Open Fracture Caused by *Mycobacterium setense* and *Clostridium celerecrescens*

**DOI:** 10.3390/antibiotics11091254

**Published:** 2022-09-15

**Authors:** Lenka Ryskova, Jan Zahradnicek, Rudolf Kukla, Radka Bolehovska, Milan Vajda, Ivo Pavlik, Pavel Bostik, Pavel Ryska

**Affiliations:** 1 Institute of Clinical Microbiology, Faculty of Medicine in Hradec Kralove, Charles University and University Hospital, 50005 Hradec Kralove, Czech Republic; 2 Department of Surgery, Faculty of Medicine in Hradec Kralove, Charles University and University Hospital, 50005 Hradec Kralove, Czech Republic; 3 Department of Diagnostic Radiology, Faculty of Medicine in Hradec Kralove, Charles University and University Hospital, 50005 Hradec Kralove, Czech Republic; 4 Faculty of Regional Development and International Studies, Mendel University in Brno, tr. Generala Piky 7, 61300 Brno, Czech Republic

**Keywords:** nontuberculous mycobacteria, rapidly growing mycobacteria, post-traumatic osteomyelitis, fracture-related infection

## Abstract

Infections caused by *Mycobacterium setense* or *Clostridium celerecrescens* are extremely rare. In this report, for the first time a dual infection with these two pathogens is described. An 18-year-old female suffered multiple injuries, including an open comminuted fracture of the right humeral diaphysis after falling from a fifth-floor balcony in January 2019. Five months after the accident, a fistula appeared in the scar, reaching the bone tissue. *M. setense* and *C. celerecrescens* were cultured from sinus swabs and subsequently from perioperative samples. The patient was initially treated with a combination of intravenous antibiotics (ATBs): imipenem, amikacin, and ciprofloxacin. One month after the fracture fixation with a titanium nail, *C. celerecrescens* was again detected; therefore, metronidazole was added to the therapy. A triple combination of oral (PO) ATBs (trimethoprim–sulfamethoxazole, moxifloxacin, and metronidazole) followed, 8 weeks after the initial intravenous therapy. *C. celerecrescens* was cultured again two times, most recently in November 2019, when surgical debridement was supplemented by the topical administration of cancellous bone impregnated with vancomycin. Signs of bone healing were found at follow-ups and ATB treatment was finished in March 2020 after a total of 9 months of therapy. To this day, there have been no signs of reinfection. This case thus illustrates the need for a combination of systemic and individualized local therapy in the treatment of complicated cases of dual infections with rare pathogens.

## 1. Case Report

In this report, a case of an 18-year-old female who suffered multiple injuries in January 2019 after falling from a fifth-floor balcony is described. The injuries included liver and spleen laceration; pulmonary contusion; comminuted fractures of the maxillary sinus, orbit, and pubic bone; open comminuted fracture of the femoral diaphysis; and an open comminuted fracture of the right humeral diaphysis. The initial treatment consisted of liver suture, splenectomy, and the external fixation of open humerus and femur fractures. After a week, definitive osteosynthesis of femur and humerus was performed (using a metaphyseal plate) and, in March 2019, the patient was discharged from the hospital.

**Figure 1:**
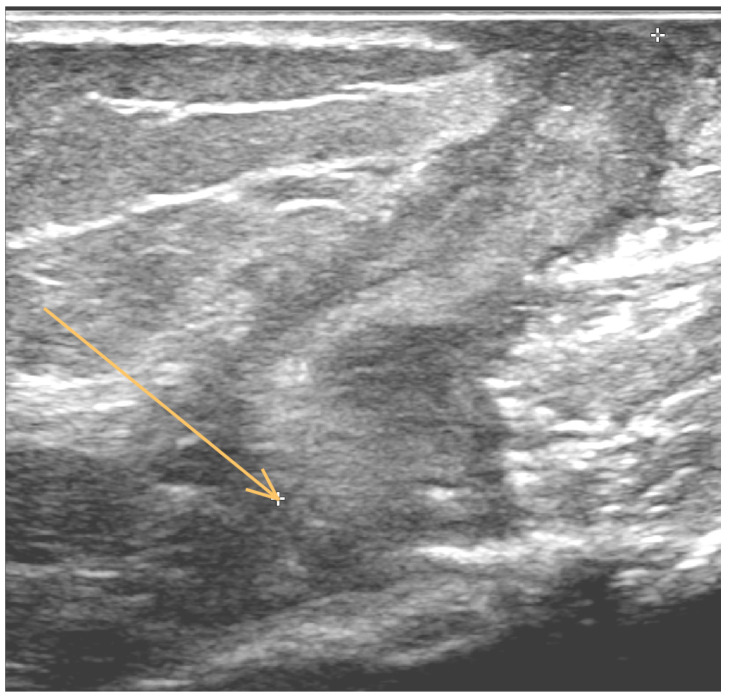
Ultrasound examination of the proximal part of the right arm shows a fluid-thickened collection (indicated by arrow).

In June 2019, a small fistulous defect with secretion appeared in the scar on the right arm, accompanied by a painful edema. Ultrasound examination of the proximal part of the right arm showed a fluid-thickened collection reaching the bone (Figure 1). The patient was afebrile with CRP of 42 mg/L (normal range 0–5 mg/L). A sinus swab was performed and transported to the Department of Clinical Microbiology (University Hospital Hradec Kralove, Hradec Kralove, Czech Republic) for culture. After 4 days of culture under aerobic conditions at 37 °C on blood agar, small rough colonies were observed. Gram and Ziehl–Neelsen staining revealed the formation of irregular gram-positive and acid-fast rods, respectively. The isolate was identified as *Mycobacterium setense* (*M. setense*) using the MALDI-TOF MS (Bruker Daltonics GmbH, Bremen, Germany). After 2 days of an anaerobic culture at 37 °C, large colony-forming units (CFU) grew on Schaedler agar, which were identified as *Clostridium celerecrescens* (*C. celerescens*) by MALDI-TOF MS. Because of the rarity of these two pathogens acting as an infectious agent, both microbes were subsequently confirmed by molecular genetic methods.

Antibiotic (ATB) susceptibility examination was performed by the E-test and broth microdilution methods (Table 1).

**Figure 2:**
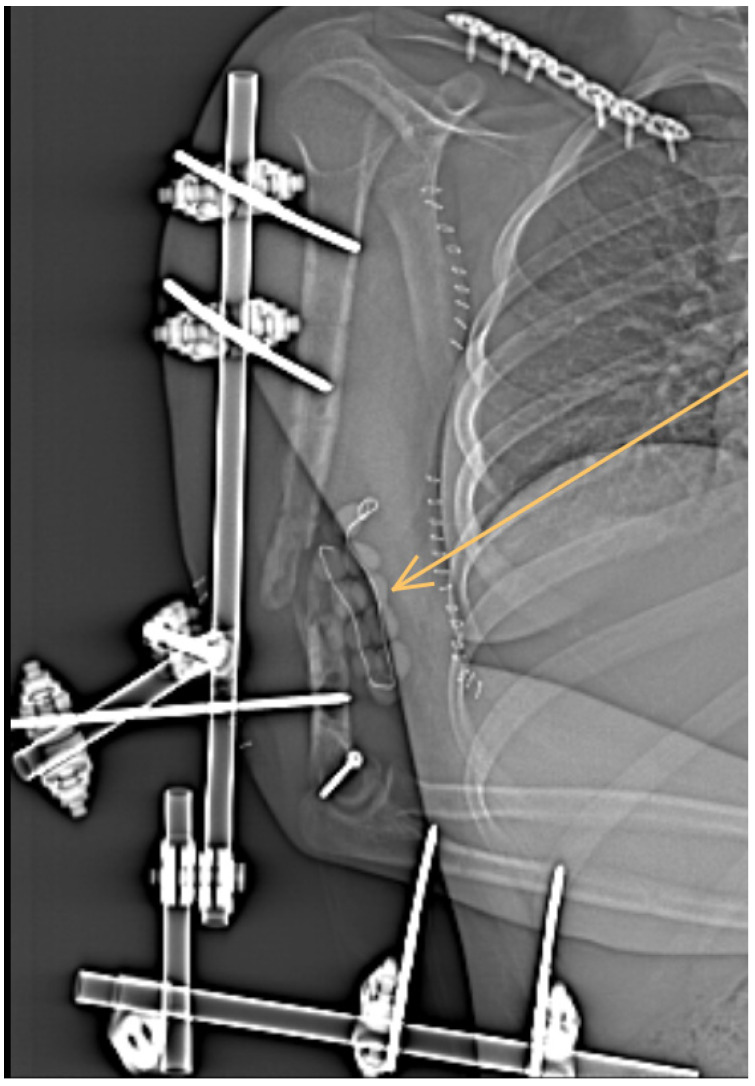
The fracture was then stabilized by an external fixation and the affected area was filled with gentamicin polymethylmethacrylate beads (indicated by arrow).

During the surgical revision, no signs of healing were found. Moreover, the site of fracture was filled with a necrotic mass and pus, which were visible under the entire osteosynthetic plate. Debridement of the wound was performed, the necrotic mass was removed, and the affected area was filled with gentamicin polymethylmethacrylate beads (Septopal, Biomet-Merck, Bridgend, UK). The fracture was then stabilized by an external fixation (Figure 2).

Two samples of the visibly infected tissue and the removed osteosynthetic plate were sent for cultivation, during which both bacterial species, i.e., *M. setense* and *C. celerecrescens,* were again confirmed in all collected samples. After the surgery, a combination of intravenous (IV) ATB (selected to be effective against both species) was used: imipenem 1 g per 6 h, amikacin 1 g once a day, and ciprofloxacin 400 mg every 12 h. After a month (18 July 2019), surgical revision was needed. The bone was not healed due to infection and another debridement was performed. Further stabilization was needed, so the external fixation was removed and a titanium plate for internal fixation was applied. Metronidazole 500 mg IV every 8 h was added to the antibiotic therapy regimen. The ATB treatment was continued for another 4 weeks.

**Figure 3:**
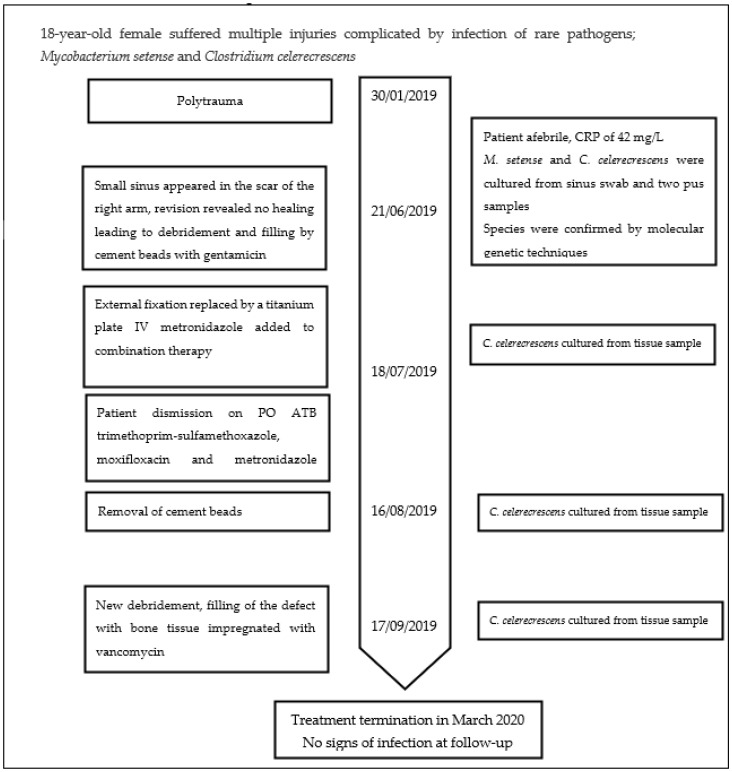
The timeline of the case developments.

After a total of 8 weeks of IV therapy, the patient was discharged and a triple combination of PO ATBs (trimethoprim–sulfamethoxazole 1440 mg every 12 h, moxifloxacin 400 mg once a day, and metronidazole 500 mg every 8 h) was initiated. On 17 September 2019, after an elective removal of the cement beads (Septopal), *C. celerecrescens* was cultured again from a tissue sample. On 1 November 2019, the debridement of necrotic mass and the subsequent filling of the defect with cancellous bone were performed. The bone tissue used was presurgically impregnated in a vancomycin solution for 30 min. After the revision surgery, *C. celerecrescens* was cultured again. A triple combination of PO ATBs continued afterward and during the regular follow-up, and signs of bone healing were found. The treatment was finished in March 2020 and, until today, no signs of reinfection were detected (Figure 3).

## 2. Literature Review and Discussion

This case report describes for the first time an occurrence of a difficult-to-treat dual infection in the site of an open fracture caused by two rarely identified pathogens, *M. setense* and *C. celerecrescens*. Fracture-related infections (FRIs) occur in between 1 and 30% of fractures, depending on the severity of the injury and initial management, and higher incidence is mainly associated with open fractures [1]. FRIs are generally caused by direct contamination of the wound due to the trauma, during insertion of the fixation device, or during impaired wound healing [2]. Common causative agents of FRIs include *Staphylococcus aureus*, coagulase-negative staphylococci, enterobacteria, *Pseudomonas aeruginosa*, streptococci, and anaerobes. The prevalence of individual pathogens varies depending on whether it is an early, delayed, or late infection [2,3]. Causative agents usually originate in the external environment, the patient’s own microflora, or a hospital environment. Open fractures potentially lead to wound contamination with soil microorganisms. In this type of FRI, Enterococci and *Enterobacterales* are frequent etiological agents. [4]. Both types of microbes, which are otherwise cultured rarely in our laboratory (unpublished data), are found in the soil and are usually associated with late post-traumatic infections [5,6].

*M. setense* is a rapidly growing mycobacterium (RGM) belonging to a large group of nontuberculous mycobacteria (NTM), with CFU being observed within a maximum of 7 days also growing on blood-enriched culture media. It is a member of the *M. fortuitum* complex rarely isolated from humans, animals, and the environment [7]. *M. setense* was first described in 2008 in a patient suffering from a post-traumatic chronic soft-tissue infection and osteitis of the foot, which developed after stepping on a nail. Initially, the isolate was identified as *M. peregrinum* and thus it was treated with clarithromycin, not surprisingly without success due to the common resistance to this ATB. Subsequently, a multigene sequencing approach was performed and a new species of *M. setense* was discovered and named after the place of origin, the city of Sete, France [5].

In the same year, another strain of *M. setense* was isolated from pus in a maxillary cavity after a bone graft implantation in this area [8]. This isolate was also resistant to macrolides. The infection was initially successfully treated with a combination of imipenem and ciprofloxacin IV, followed by a PO administration of ciprofloxacin for 3 months. In 2017, *M. setense* was isolated from infected aquarium fish in Slovenia [9] and Italy [10]. In 2021, panniculitis in a cat caused by *M. setense* was described in Germany. Infection was treated for 33 months without complete recovery. Therapy was also complicated by the development of resistance to the administered fluoroquinolone [11].

In human patients *M. setense* was rarely isolated in different countries, e.g., in Asia [12,13] and France [5,14]. In the environment, this NTM species was found in Iran in various samples from hospitals, e.g., a water system [15,16], soil and dust [17] and the environment [18].

Although there is only a limited number of studies on *M. setense* infections and its susceptibility to antibiotics, this species appears to be characteristically resistant to macrolides [19,20], a feature also observed in the described case (Table 1).

*C. celerecrescens* was first isolated from cow dung in 1989, and the name *celerecrescens* was derived from its rapid growth [21]. Case reports of post-traumatic osteomyelitis caused by this *Clostridium* have been presented in the literature only five times so far [6], with the first report in 2005 describing an open femoral fracture infection [22]. Another case was described by Mischnik et al. [23] in a 55-year-old male with an open tibial fracture, in whom osteomyelitis developed at the site of the original fracture 9 years after the injury, with *Staphylococcus aureus* and *C. celerecrescens* co-infections as proven agents. The therapy in this case consisted of surgical intervention and administration of cefuroxime and metronidazole for 8 weeks. Ten months later, the patient was admitted with similar symptoms and *C. celerecrescens* was repeatedly cultured. Therefore, the identical combination of ATBs for 6 weeks was used to successfully treat the infection [23]. A complicated course of infection, where *C. celerecrescens* was repeatedly isolated from perioperative samples despite continuous ATB therapy for 7 months, was described by Mormeneo Bayo et al. [6]. In the case described in this report, the treatment of the infection was also challenging, since *C. celerecrescens* was repeatedly cultured from the site of infection during the period between June to November 2019, despite adequate antimicrobial therapy.

In general, the treatment of fracture-related infections is difficult, and the treatment of late infections accompanied by biofilm formation is even more complicated. Therefore, in addition to surgical treatment, a diagnostic approach ensuring the identification of an etiological agent and susceptibility examination, or at least the knowledge of the ATB profile necessary for targeted ATB therapy, is important. Prolonged culture of deep tissue samples, including the extracted osteosynthetic material, for at least 7–10 days is strongly recommended for the bacteriological detection of the causative agent [2]. Under these conditions, RGM can be detected, although their involvement is usually not anticipated. The recommended length of the subsequent ATB therapy varies depending on whether the fixation material can or cannot be removed. Therapy for 6 weeks is considered sufficient if the removal is possible, but 12-week treatment is otherwise recommended [2]. Longer therapy is advocated for mycobacterioses affecting the musculoskeletal system, lasting usually at least 6 months with an extension of up to 12 months in more complicated and severe infections [24,25]. In the case of the patient described herein, the treatment required a total of 9 months of ATB therapy.

However, such systemic antimicrobial therapy may not be sufficient, and a local ATB application may be necessary to potentiate the effects of the therapy. For a long time now, the bone cement beads augmented with gentamicin are used as local ATB carriers to fill the osteomyelitic defects. A major disadvantage of this treatment is the need for subsequent surgical extraction. Thus, in this case, bone grafts saturated pre-surgically with a selected ATB, which do not need any subsequent surgical intervention, have been used. The kinetics of ATB released from the bone tissue are similar to the bone cement. In the beginning, high levels of released ATB are detected; then, in a few hours, the concentration of ATB progressively decreases until only low concentrations are released. Bactericidal concentrations can be maintained for a time range of 5 days up to several weeks depending on the type of bone graft and ATB, and the method of impregnation [26]. In the case reported here, cancellous bone saturated with vancomycin after repeated cultivation of *C. celerecrescens* was used. This therapy led to the development of the apparent healing process.

## 3. Materials and Methods

Swabs from the sinus were cultivated on blood agar, MacConkey agar and Schaedler agar (Thermo Fisher Scientific, Basingstoke, UK) at 37 °C under aerobic and anaerobic condition.

The isolates were identified as *M. setense* and *C. celerecrescens* using the MALDI-TOF MS (Bruker Daltonics GmbH, Bremen, Germany). Identification of both microbes was subsequently confirmed by molecular genetic methods. Nucleic acids were isolated directly from the suspension of bacterial isolates using the QIAamp DNA Mini Kit (Qiagen, Hilden, Germany). Amplification and sequencing of single copy gene encoding the RNA Polymerase β Subunit (*rpo*B) with primers *rpo*B-F (5′-GGCAAGGTCACCCCGAAGGG-3′) and *rpo*B-R (5′-AGCGGCTGCTGGGTGATCATC-3′) was used for *M. setense* confirmation as described by Adekambi et al. [27]. *C. celerecrescens* identification was performed by 16S rRNA region amplification and sequencing with universal primers complementary to four conserved regions alongside two hypervariable sequences V3 (5′-CCAGACTCCTACGGGAGGCAG-3′) and V6 (5′-ACATTTCACAACACGAGCTGACGA-3′). Sequences of primers were obtained from study by Chakravorty et al. [28]. The sequencing was performed using ABI 3500 Genetic Analyzer (Applied Biosystems/Thermo Fisher Scientific, Foster City, CA, USA) and final sequences were subsequently processed with Bionumerics 7.6.2 software (Applied Maths, Ghent, East Flanders, Belgium). The nucleotide sequence analysis using the NCBI BLAST and SepsiTestTM BLAST showed the best BLAST score with *C. celerecrescens* and *M. setense* (>99% identity in both databases).

Antibiotic (ATB) susceptibility examination was performed by the E-test and broth microdilution method. MIC values of penicillin, amoxicillin, clindamycin, metronidazole, vancomycin and linezolid were performed by E-test (bioMérieux, Marcy l’Etoile, France) in Mueller-Hinton agar with 5% defibrinated horse blood and 20 mg/L β-NAD (Thermo Fisher Scientific, Basingstoke, UK) and the results were interpreted according to the European Committee on Antimicrobial Susceptibility Testing (EUCAST) for *C. celerecrescens*. MIC values of clarithromycin, amikacin, imipenem, linezolid, tigecycline, ciprofloxacin, moxifloxacin, trimethoprim-sulfamethoxazole, and doxycycline were performed using the broth microdilution method with 96-well microtiter plates. The cation-adjusted Mueller-Hinton (Becton Dickinson, Sparks, MD, USA) was used and the results were interpreted according to the Clinical and Laboratory Standards Institute [29] for *M. setense*. Only those antibiotics with predicted antimicrobial effect against anaerobes and mycobacteria were used for susceptibility tests.

## 4. Conclusions

*M. setense* and *C. celerecrescens* are rare pathogens capable of causing early and deep infections, mostly of post-traumatic origin, due to wound contamination by different environmental matrices. In the presented case, polymicrobial FRI infection was managed using a comprehensive approach that involved repeatedly performed surgical debridement and a systemic and local administration of ATB based on the establishment of the MIC of causative pathogens. This approach led to the successful management of the infection and subsequent healing.

## Figures and Tables

**Table 1:** Susceptibility profile and MIC values (mg/L) of *Mycobacterium setense* and *Clostridium* *celerecrescens*.

*Mycobacterium setense*			*Clostridium celerecrescens*		
Antimicrobial	MIC	Interpretation	Antimicrobial	MIC	Interpretation
Clarithromycin	24	R	Penicillin	0.19	S
Amikacin	0.38	S	Amoxicillin	0.25	S
Imipenem	0.5	S	Clindamycin	12	R
Linezolid	12	R	Metronidazole	0.047	S
Tigecycline	0.032	S	Vancomycin	0.5	S
Ciprofloxacin	0.032	S	Linezolid	0.16	S
Moxifloxacin	0.064	S	Imipenem	0.25	S
Trimethoprim–sulfamethoxazole	0.125	S			
Doxycycline	24	R			

MIC = Minimum inhibitory concentration, S—susceptible, R—resistant; results interpretation based on breakpoints by CLSI (*M. setense*) and EUCAST (*C. celerecrescens*).

## Data Availability

Data are available on request due to the ethical restrictions.
